# Antibiotic treatment and supplemental hemin availability affect the volatile organic compounds produced by *P.* *gingivalis* in vitro

**DOI:** 10.1038/s41598-022-26497-0

**Published:** 2022-12-29

**Authors:** Kajsa Roslund, Moona Uosukainen, Katriin Järvik, Kari Hartonen, Markku Lehto, Pirkko Pussinen, Per-Henrik Groop, Markus Metsälä

**Affiliations:** 1 Department of Chemistry, University of Helsinki, Helsinki, Finland; 2 Folkhälsan Institute of Genetics, Folkhälsan Research Center, Biomedicum Helsinki, Helsinki, Finland; 3 Abdominal Center Nephrology, University of Helsinki and Helsinki University Hospital, Helsinki, Finland; 4 Clinical and Molecular Metabolism, Faculty of Medicine Research Programs, University of Helsinki, Helsinki, Finland; 5 Oral and Maxillofacial Diseases, Helsinki University Hospital, University of Helsinki, Helsinki, Finland; 6 Institute of Dentistry, University of Eastern Finland, Kuopio, Finland; 7 Department of Diabetes, Central Clinical School, Monash University, Melbourne, VIC Australia

**Keywords:** Biosynthesis, Metabolic pathways, Mass spectrometry, Medical and clinical diagnostics, Antimicrobials, Applied microbiology, Pathogens, Bacterial toxins, Diagnostic markers, Periodontitis

## Abstract

We have measured the changes in the production of volatile organic compounds (VOCs) by the oral pathogen *Porphyromonas* *gingivalis*, when treated in vitro with the antibiotic amoxicillin. We have also measured the VOC production of *P.* *gingivalis* grown in the presence and absence of supplemental hemin*.* Planktonic bacterial cultures were treated with different amounts of amoxicillin in the lag phase of the bacterial growth. Planktonic bacteria were also cultured with and without supplemental hemin in the culture medium. Concentrations of VOCs were measured with proton-transfer-reaction time-of-flight mass spectrometry (PTR-ToF–MS) and further molecular identification was done with gas chromatography–mass spectrometry (GC–MS) using solid phase microextraction (SPME) for sampling. The cell growth of *P.* *gingivalis* in the cultures was estimated with optical density measurements at the wavelength of 600 nm (OD_600_). We found that the production of methanethiol, hydrogen sulfide and several short- to medium-chain fatty acids was decreased with antibiotic treatment using amoxicillin. Compounds found to increase with the antibiotic treatment were butyric acid and indole. In cultures without supplemental hemin, indole and short- to medium-chain fatty acid production was significantly reduced. Acetic acid production was found to increase when supplemental hemin was not available. Our results suggest that the metabolic effects of both antibiotic treatment and supplemental hemin availability are reflected in the VOCs produced by *P.* *gingivalis* and could be used as markers for bacterial cell growth and response to threat. Analysis of these volatiles from human samples, such as the exhaled breath, could be used in the future to rapidly monitor response to antibacterial treatment.

## Introduction

*Porphyromonas* *gingivalis* is a gram-negative oral bacteria involved in the development and deterioration of periodontitis, an inflammatory disease destroying the soft and bone tissues supporting the teeth. Periodontitis is a risk factor for many other systemic diseases and the presence of *P.* *gingivalis* has been shown to correlate with the occurrence of diabetes, Alzheimer’s disease, cardiovascular diseases, cancer, and adverse pregnancy outcomes^1^. Often these diseases exacerbate each other in a vicious cycle, with the oral inflammation leading to deteriorating systemic health, which leads to weakened immune response and more inflammation^2^. *P.* *gingivalis* is also connected to dental abscesses, which can cause sepsis if large amounts of bacteria enter the bloodstream^3^. Initial to severe periodontitis, including stages I-III is recommended to be treated with oral cleaning, such as scaling or smoothening of the root surfaces, without the routine use of systemic antibiotics or adjunctive therapy^4^. Advanced periodontitis, however, may require surgical procedures, such as grafting or removal of flaps, as well as antibiotics^5^. However, the overuse of antibiotics and the increasing antibiotic resistance has become one of the greatest problems of the modern society. New, more specific ways to target bacterial infections are developed constantly to battle this, but the high cost and poor availability of such treatments encourage the use of traditional antibiotics, especially in the developing countries. Fast identification of pathogens is vital for a targeted treatment. Traditionally this is done via culturing or polymerase chain reaction (PCR) methods, which can be time consuming. This can either prolong the treatment or encourage the use of broad-spectrum antibiotics. In addition, the progression of the antibiotic treatment is usually monitored only by evaluating the patient’s symptoms. A method for fast identification and monitoring of a bacterial infection could help reduce the unnecessary use of broad-spectrum antibiotics, while ensuring the success of the treatment. Analysis of volatile organic compounds (VOCs) produced by bacteria has been suggested as a fast method for identifying pathogens from samples such as exhaled breath and blood, urine, or salivary headspaces^6–12^.

Amoxicillin is a broad-spectrum, penicillin antibiotic, which inhibits the cell wall biosynthesis of bacteria. It is one of the most used antibiotics for oral infections and advanced periodontitis, commonly combined with a β-lactam drug to overcome resistance^13^. It is also used in combination with other antibiotics, such as metronidazole, to enhance the antimicrobial effect^5,13^. It is commonly acknowledged and shown in vitro and in vivo that amoxicillin, in combination with a β-lactam drug or metronidazole, is extremely effective against periodontal pathogens^5,13–15^. However, there is also evidence that many periodontal pathogens show resistance to amoxicillin^16,17^, penicillin antibiotics alone have no significant effect on the periodontal microbiota^18^, and that sublethal amoxicillin concentrations can increase the biofilm formation^19^. Outside of proteomics and gene expression research, studies investigating the effects of antibiotics on the metabolites of *P.* *gingivalis* are scarce, and those studying the effect of antibiotics on volatile metabolites are even more so. However, studies on the effects of antibiotics and other environmental factors on bacterial metabolites exist for some species, such as *Escherichia* *coli,* *Mycobacterium* *smegmatis,* *Staphylococcus* *aureus*, and *Pseudomonas* *aeruginosa*^20–29^. Extracellular hemin is an important environmental factor for the normal metabolism of *P.* *gingivalis*^30^, and in hemin-poor conditions *P.* *gingivalis* exhibits decreased virulence^31^. Liquid-phase studies investigating the effects of hemin on the metabolites of *P.* *gingivalis* have been performed^31^, but no studies exist for volatile metabolites. If antibiotic treatment or an environmental factor changes the VOC-profile of *P.* *gingivalis* in vitro, changes may also be seen in the VOC-profiles of human samples in vivo. This could open new possibilities for monitoring the response of bacterial infections to antimicrobial treatment.

We have previously identified some of the most important VOCs produced by oral pathogenic bacteria, including *P.* *gingivalis*^6,7^. These include several sulfur compounds, such as methanethiol, hydrogen sulfide, 2-methylpropanethiol, dimethyl disulfide, dimethyl trisulfide, dimethyl tetrasulfide, and *S*-methyl thioesters, as well as several short- and medium-chain fatty acids, several isoamyl esters, 6-methyl-1,2,3,4-tetrahydroquinoline, and 1-methyl-1,2,3,4-tetrahydroisoquinoline^6,7^. In the current study, we wanted to examine whether the changes in environmental factors have an effect on the VOCs produced by *P.* *gingivalis* and whether these changes could be used to evaluate the viability or virulence of the bacteria. We chose hemin and amoxicillin as environmental variables because their effects on the metabolism of *P.* *gingivalis* in general are relatively well documented, although their effects on its VOC production are still unknown. We used a combination of proton-transfer-reaction time-of-flight mass spectrometry (PTR-ToF–MS) and solid phase microextraction (SPME) followed by gas chromatography–mass spectrometry (GC–MS) to identify and measure the concentrations of VOCs. We examined the changes in the volatiles produced by planktonic cultures of *P.* *gingivalis* in the presence of amoxicillin and when supplemental hemin was not available. Our aim was to identify compounds that were most significantly affected by amoxicillin and the availability of supplemental hemin, and to evaluate their potential as markers for bacterial response to antimicrobial treatment. In this article, we also discuss the possible metabolic origins for the changes in the VOC-production of *P.* *gingivalis*.

## Materials and methods

### Initial bacterial cultures

The reference strain of *P.* *gingivalis* ATCC 33277 (serotype Pg a) was acquired from the American Type Culture Collection (ATCC). The culture method used has been described previously by us^6,7^. The reference strain was stored at − 80 °C in frozen skim milk. Strain was activated by streaking onto the Brucella blood agar (BBLTM, 211086) plates, supplemented with horse blood (5% v/v), hemin (5 mg/L) and vitamin K (1 mg/L). The initial culture was incubated in an anaerobic gas mixture (5% CO_2_, 10% H_2_ and 85% N_2_) at 37 °C for 72 to 120 h. After the incubation, 3.0 mL of phosphate-buffered saline (PBS) was pipetted onto the agar plate and the bacterial mass was gently scraped from the agar into a 5 mL Falcon™ tube. The resulting bacterial suspension was homogenized by gently pipetting. From this bacterial suspension 500 µL was pipetted into 5.0 mL of Tryptic soy broth (TSB, ATCC 2722) supplemented with hemin (5 mg/L) and vitamin K (1 mg/L). This bacterial culture was incubated anaerobically without stirring at 37 °C until the optical density at 600 nm reached 1.0 (batch 0). This corresponds to roughly 10^9^ cells/mL.

### Negative and positive controls

Negative control used in this study was fresh TSB (ATCC 2772) without any bacteria added. Triplicates of 10 mL of TSB were prepared in 50 mL bottles for the negative control measurements. Positive control was prepared with 1.25 mL of the bacterial culture described in the previous section (batch 0) added into 10 mL of fresh TSB (ATCC 2722) in a 50 mL bottle. Triplicates of positive control were also prepared.

### Antibiotic treatment

Amoxicillin (Sigma Aldrich) was dissolved in minimal amount (one to two drops) of 1 M NaOH solution. The final concentrations of amoxicillin used in the measurements were diluted from this batch with sterilized water. Bacterial growth curves measured using OD_600_ were used to estimate the lag, exponential, and stationary phases of bacterial growth. Time points of 0, 15 and 30 h were determined, respectively. Test concentrations of amoxicillin were added to the cultures in these time points to investigate the effects of antibiotic treatment in different stages of bacterial life cycle. Accordingly, the lag phase was chosen for the VOC measurements, because the effects of amoxicillin on the bacterial growth were largest. According to the growth curves in different amoxicillin concentrations, two exposure concentrations (15 and 150 µg/mL) were chosen for the VOC measurements. Triplicate cultures in 50 mL bottles were prepared for the VOC measurements by adding 1.25 mL of the bacterial culture (batch 0) into 10 mL of fresh TSB containing amoxicillin.

### Limiting supplemental hemin

First passage cultures were prepared in 50 mL bottles with 1.25 mL of bacteria (batch 0) added into 10 mL of fresh TSB not supplemented with hemin (ATCC 2722 (− hemin)). Triplicates were prepared. Bacterial growth curves measured using OD_600_ with and without supplemental hemin present can be found from the Supplementary Information (Fig. S4). No significant differences in bacterial loads were observed between cultures with and without supplemental hemin present, however, the exponential and stationary phases of the bacterial growth were reached slightly later in cultures lacking supplemental hemin.

### Measurement and identification of VOCs

The PTR-ToF–MS method and operation conditions have been previously described by us^6,7^. Measurements were performed from *m/z* 17 to 239, with H_3_O^+^ as the reagent ion. The mass resolution of the instrument used (PTR-TOF 1000, Ionicon) is 1500 (full-width at half-maximum). PTR-ToF–MS operating conditions were as follows: reduced electric field (*E/N*) of 116 Td (corresponding to drift tube voltages of 500 V); drift tube pressure of 2.20 mbar; H_2_O flow of 5.0 standard cubic centimeters per minute (sccm); ion source current of 3.0 mA; inlet flow of 65 sccm. Drift tube and inlet temperatures were kept at 70 °C. Sampling frequency was 1 spectrum/s. In addition, a separate sensor for the measurement of CO_2_ (Vaisala, GMP251) was used. To obtain accurate concentrations, the PTR-ToF–MS instrument should be calibrated separately for each individual compound, which in the case of bacterial samples are numerous. In this study, the PTR-ToF–MS instrument was not calibrated specifically for each analyzed compound, and therefore, the concentrations presented in this work should be considered estimates.

The GC–MS method and operation conditions have also been previously described by us^7^. Briefly, the GC-instrument (Agilent 6890 A) using electron ionization (EI) combined with a quadrupole mass spectrometer (Agilent 5973N MSD) was used. The column (DB-1701, J&W Scientific) length, inner diameter and film thickness were 30 m, 0.25 mm, and 0.15 µm respectively. Temperature was ramped up after 2 min hold from 40 °C to 250 at 10 °C/min. Splitless injection, with splitless time of 2 min from injection, was used. Injection port was kept at 240 °C. Carrier gas (99.996% helium, Linde Gas) the flow rate was 1.0 mL/min. GC–MS transfer line was kept at 250 °C. Measurements were performed in the mass scan range of 20 to 300 u. The ionization energy, ion source temperature and quadrupole temperature were 70 eV, 230 °C and 150 °C, respectively. Volatiles from the headspace of the bacterial cultures were sampled with a polydimethylsiloxane/divinylbenzene SPME Arrow (CTC Analytics AG, Zwingen, Switzerland). The National Institute of Standards and Technology NIST14 Mass Spectral Library and Analysis Tools were used for the tentative identification of compounds from the measured mass spectra. Most compounds were also further identified using reference samples (Sigma Aldrich).

**Figure 1:**
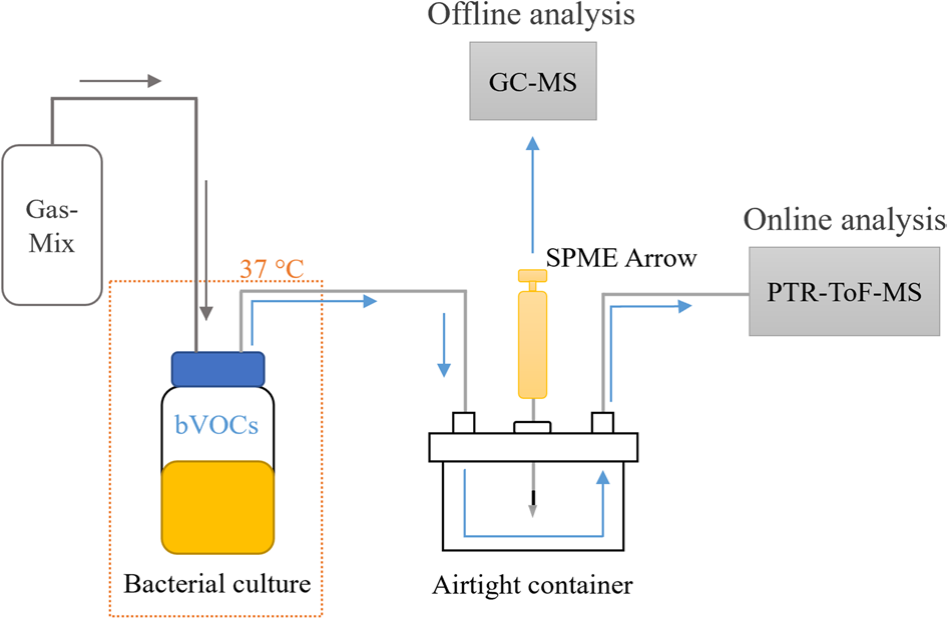
Schematic representation of the sampling process. Anaerobic gas-mixture flows through the bacterial culture headspace into a container, where the SPME sample is taken, and excess moisture is collected. PTR-ToF–MS instrument measures the concentrations of VOCs in the headspace and GC–MS is used for their identification. Bacterial cultures are incubated at 37 °C, but otherwise the setup is in room temperature.

Figure 1 describes the complete setup used in this study for bacterial VOC measurements with PTR-ToF–MS and GC–MS. PTR-ToF–MS measurements were made continuously for 100 h, with each of the triplicates measured for 20 min at a time. A flow of 70 sccm of the anaerobic gas-mix was directed through the bacterial cultures. Samples for GC–MS measurements were taken at 0 and 100 h and analyzed offline. Culture conditions were kept at 37 °C and atmospheric pressure during the measurement.

Statistical evaluation was performed by calculating the mean concentration and standard deviation of triplicate samples (N = 3).

## Results

Examples of growth curves for *P.* *gingivalis* in different amoxicillin concentration, as well as the bactericidal performance of amoxicillin in different phases of the bacterial growth, are presented in the Supplementary Information (Fig.S1). Examples of growth curves for *P.* *gingivalis* cultured with and without supplemental hemin present are also presented in the Supplementary Information (Fig. S4). A complete list of the compounds identified from the headspace of *P.* *gingivalis* with GC–MS, excluding compounds produced by the TSB, is presented in the Supplementary Information (Table S1). The corresponding PTR-MS signals, as well as theoretical protonated masses are also presented in Table S1. An example of a VOC only produced by the TSB, but not by *P.* *gingivalis*, is also presented in the Supplementary information (Fig. S3).

**Figure 2:**
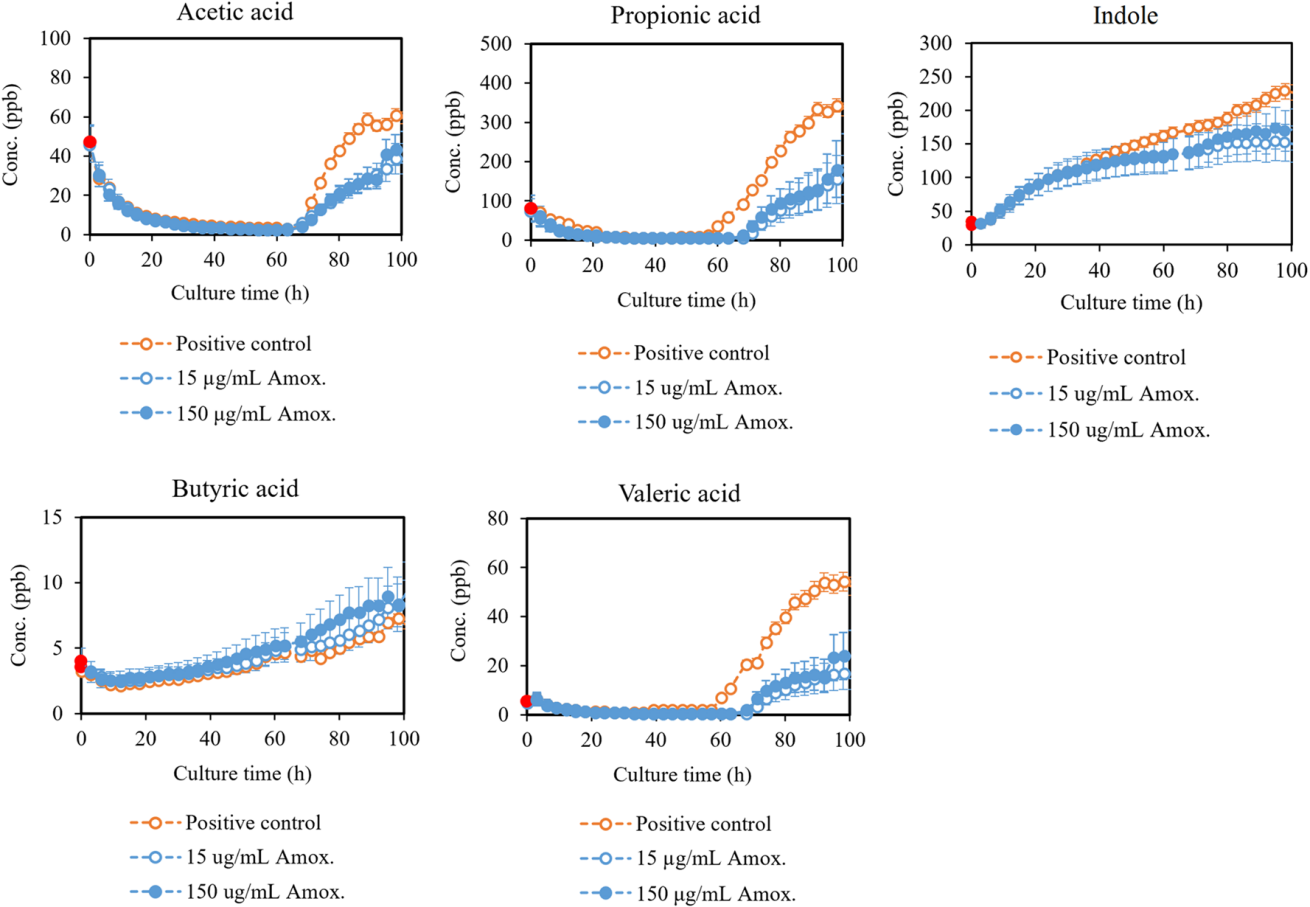
Most important volatile short- and medium-chain fatty acids and indole produced by *P.* *gingivalis* untreated (positive control) and treated with amoxicillin. The time of antibiotic addition is marked in red. Concentrations are given as the mean ± SD of triplicate measurements (N = 3). Additional production profiles (caproic, 2-methylhexanoic, and caprylic) can be found in the Supplementary information (Fig. S2).

**Figure 3:**
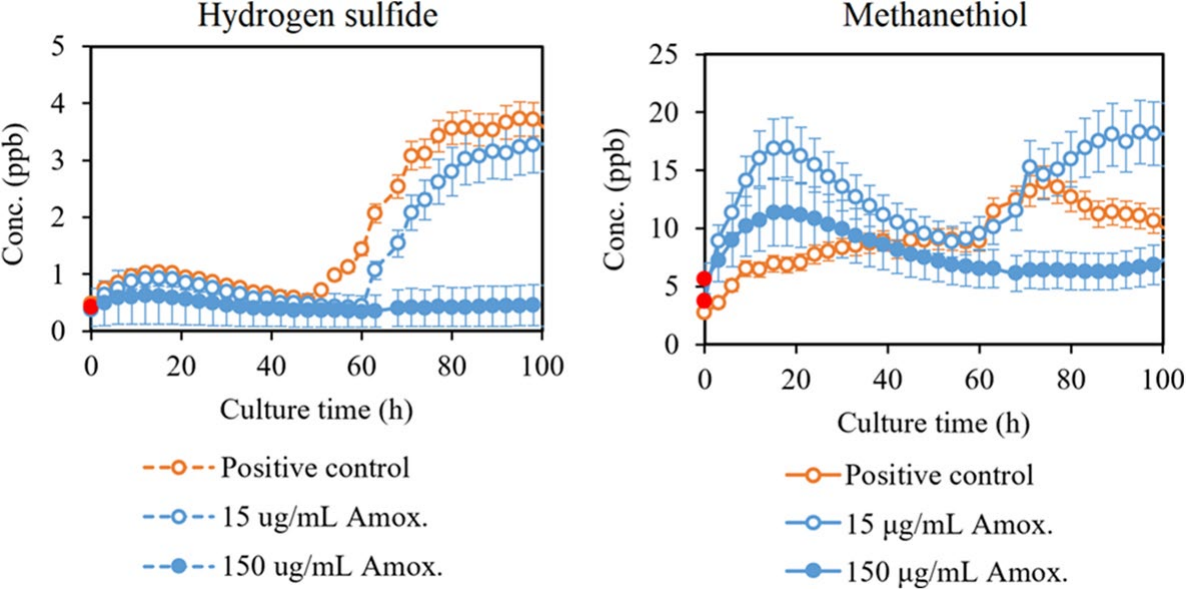
Hydrogen sulfide and methanethiol produced by *P.* *gingivalis* untreated (positive control) and treated with amoxicillin. The time of antibiotic addition is marked in red. Concentrations are given as the mean ± SD of triplicate measurements (N = 3).

Here we present production profiles for VOCs significantly affected by the antibiotic treatment and supplemental hemin availability. Figure 2 presents the total effect of amoxicillin on volatile short- and medium-chain fatty acids and indole, and Fig. 3 on methanethiol and hydrogen sulfide, when the antibiotic is added at the beginning of the culturing. In addition, the growth-corrected endpoint (90 h) concentrations (Conc. (ppb)/OD_600_) of these compounds are presented in Table 1.

**Table 1:** Endpoint concentrations of short- and medium-chain fatty acids, nitrogen and sulfur compounds corrected with the corresponding OD_600_ value. Concentrations are given as the mean ± standard deviation (SD) of triplicate measurements (N = 3). Positive control is *P.* *gingivalis* untreated with amoxicillin.

Compound	Conc. (ppb)/OD_600_ at 90 h		
	Positive control	15 µg/mL Amox	150 µg/mL Amox
Acetic acid	27 ± 3.0	14 ± 3.2	18 ± 3.0
Propionic acid	138 ± 10	56 ± 6.0	76 ± 6.4
Butyric acid	2.7 ± 0.6	3.2 ± 0.5	5.2 ± 0.5
Valeric acid	23 ± 3.2	6.9 ± 0.8	10 ± 1.0
Hydrogen sulfide	1.6 ± 0.5	1.3 ± 0.3	0.3 ± 0.3
Methanethiol	5.2 ± 0.9	8.6 ± 1.1	3.9 ± 0.5
Indole	95 ± 6.2	73 ± 6.0	106 ± 11

**Figure 4:**
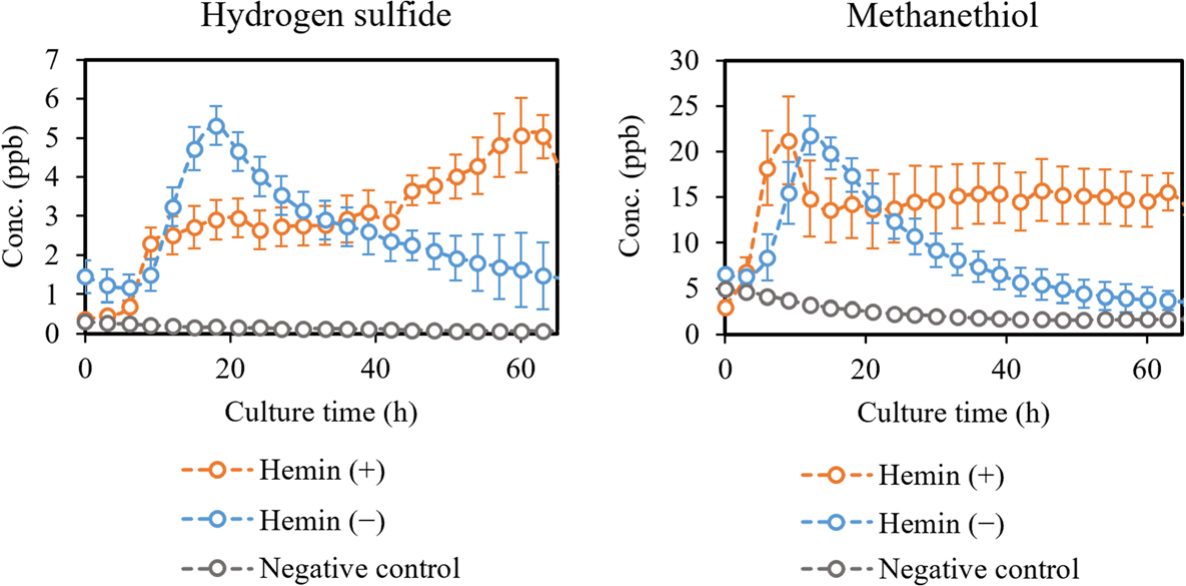
Hydrogen sulfide and methanethiol produced by *P.* *gingivalis* cultures with ( +) and without ( −) supplemental hemin added. Concentrations are given as the mean ± SD of triplicate measurements (N = 3).

**Figure 5:**
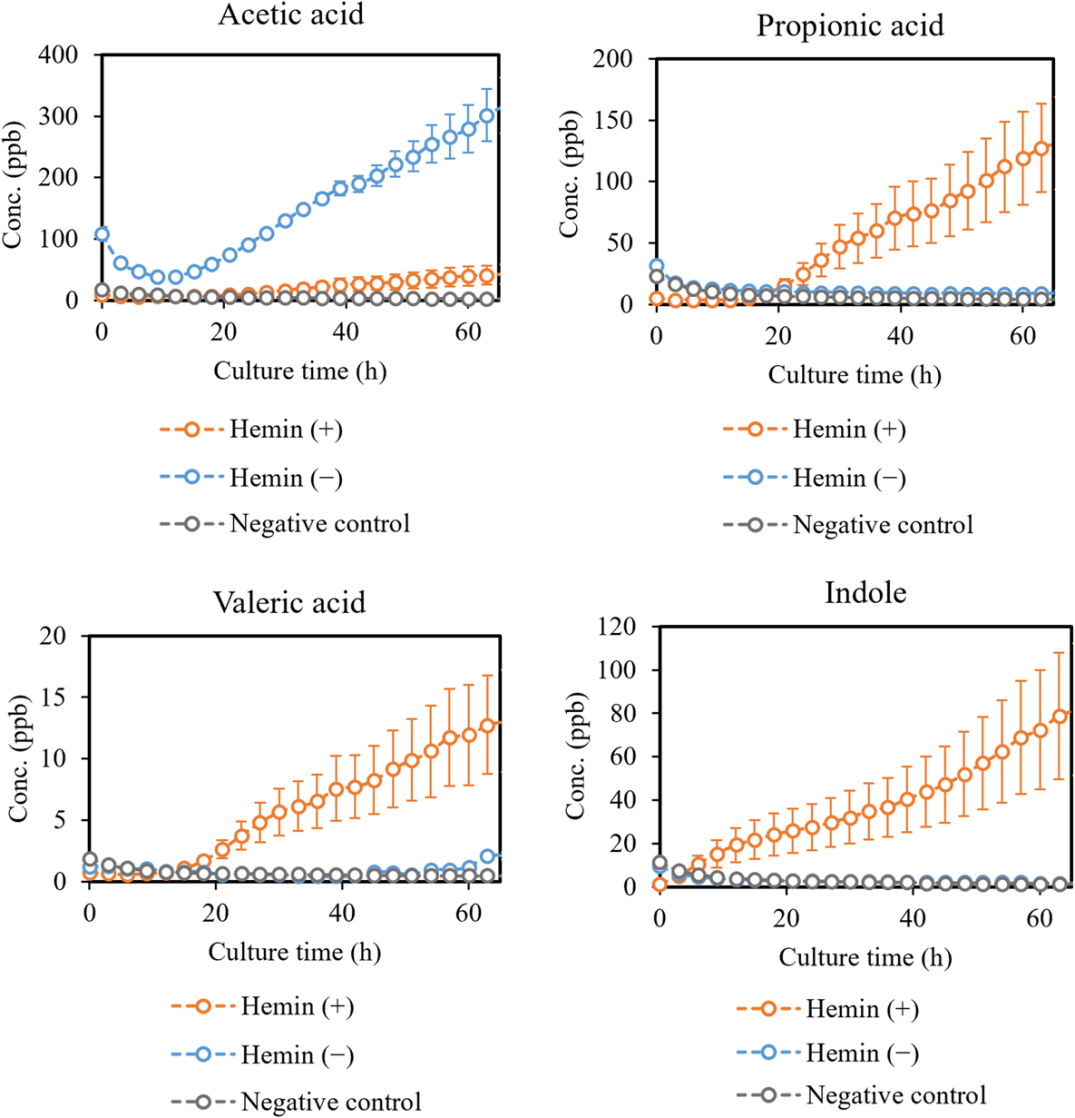
Compounds produced in increased (acetic acid) and decreased (propionic acid, valeric acid and indole) amounts by *P.* *gingivalis* cultures with ( +) and without ( −) supplemental hemin added. Concentrations are given as the mean ± SD of triplicate measurements (N = 3).

Figure 4 presents the effects of supplemental hemin availability on sulfur compounds hydrogen sulfide and methanethiol. Figure 5 presents the effects of supplemental hemin availability on selected short- and medium-chain fatty acids and indole. No significant differences were found in the growth of *P.* *gingivalis* with and without supplemental hemin (Supplementary Information Fig. S4), and therefore, no growth-correction was made.

## Discussion

### Effects of amoxicillin

As mentioned, studies investigating the effects of antibiotics on the metabolic products of *P.* *gingivalis* are scarce. However, studies have been done on other species, such as *E.* *coli,* *M.* *smegmatis,* *S.* *aureus*, and *P.* *aeruginosa*^20–29^, some of which investigate volatile metabolites. However, the use of different antibiotics and analysis techniques hinder the comparison between results. In addition, many of the earlier studies concentrate on separating antibiotic resistant and non-resistant bacterial strains based on one-point VOC profiles, measured with offline methods. Only a few earlier studies investigate the real-time effects of antibiotics on volatile metabolites, examine the growth corrected concentrations, or discuss the metabolic significance of the suggested marker compounds^22,23^. In this section, we compare the findings from the most relevant earlier studies regarding effects of antibiotics on bacterial metabolites to our results with *P.* *gingivalis.*

In a liquid-phase study, Belenky et al. showed that particularly medium-chain fatty acids produced by *E.* *coli* were reduced under treatment with ampicillin, an antibiotic in the same class as amoxicillin^27^. They also found that the relative concentrations of carbohydrate and energy metabolites, such as pyruvate, were increased. Ampicillin treated bacterial cells also showed increased amino acid utilization of cysteine, lysine, methionine, and tryptophan, which suggests increase in the products of these amino acids as well. These products include hydrogen sulfide, methanethiol, indole and ammonia. Indeed, another earlier liquid-phase study by Han et al. found increased production of extracellular indole by *E.* *coli* treated with ampicillin^28^. In addition, a study by Wiesner et al. examining volatile metabolites, detected decreased production of methanethiol when *E.* *coli* and *S.* *aureus* were treated with ampicillin and oxacillin, respectively^21^. Oxacillin is another antibiotic of the penicillin class. Another gas-phase study by Allardyce et al. found decreased production of several sulfur compounds, including methanethiol and hydrogen sulfide, acetic acid and indole in *E.* *coli* treated with gentamicin^25^. They also found decreased production of ammonia and dimethyl sulfide in *S.* *aureus* treated with flucloxacillin. Flucloxacillin is a penicillin class antibiotic, while gentamicin is not.

**Figure 6:**
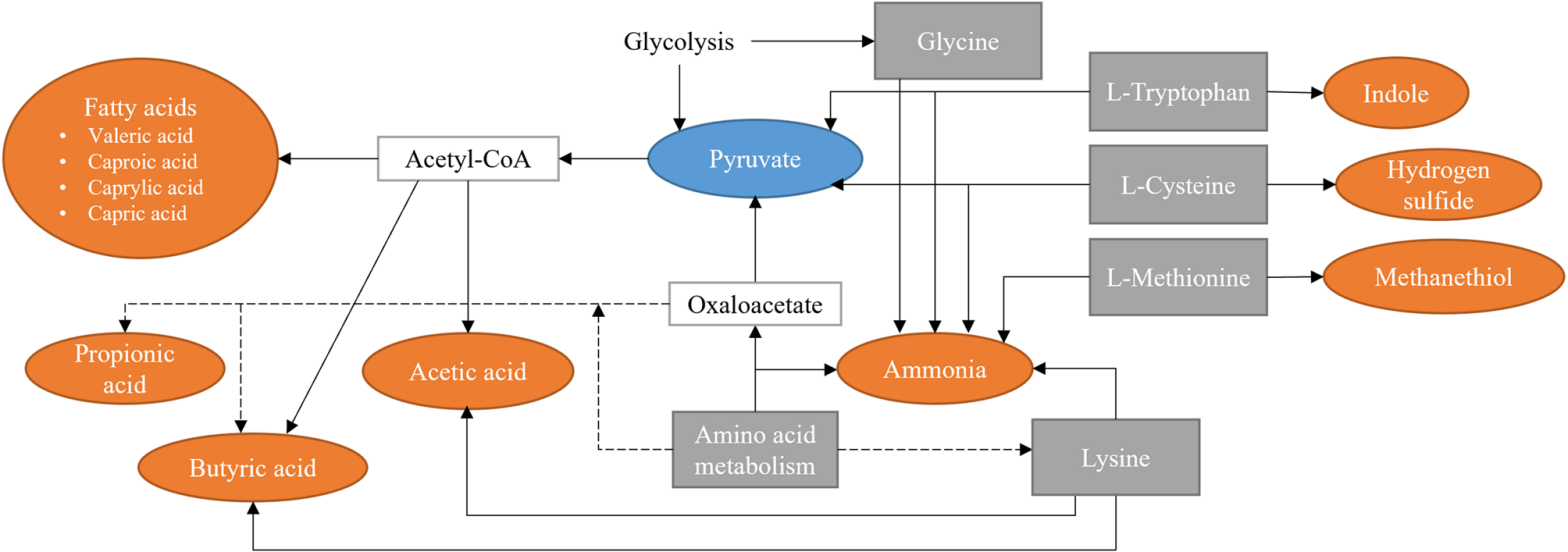
Summary of the most important metabolic routes for the formation of the VOCs found to significantly change in the presence of amoxicillin and in hemin-limited conditions. Modified from the KEGG database^38,39^ for *P.* *gingivalis* ATCC 33277 and Nelson et al.^40^. Amino acids are shown in gray, important intermediates in white, and the main volatile products in orange. Pyruvate, an important product and an intermediate is shown in blue. Dashed lines indicate multiple intermediate steps.

Our results corroborate most of the previous findings. We found that in general, amoxicillin decreases the volatile fatty acid production of *P.* *gingivalis*. These compounds include short- and medium-chain fatty acids, such as acetic, propionic, valeric, 2-methyl hexanoic and caprylic acids. Interestingly, the production of butyric acid was found to increase in the presence of amoxicillin. It was the only volatile fatty acid found to react positively to antibiotic treatment. *P.* *gingivalis* is known to produce a variety of short-chain fatty acids^31^, which have been connected both to cytotoxic as well as beneficial effects, depending on the environment^32,33^. Some of these fatty acids are beneficial especially to the gut health, while their effects in the gingival crevicular fluid of patients with chronic periodontitis are negative^34^. The concentrations seem to correlate with the severity of periodontal disease^34^. Propionic, butyric, isobutyric, valeric and isocaproic acids have also been found from the dental plaque of diseased sites^35^. Studies have also shown that many different types of cells, including oral epithelial cells, are sensitive to these acids^35,36^. It is therefore hypothesized that short-chain fatty acids are important virulence factors for periodontal pathogens causing host cell detachment, bacterial penetration, and tissue destruction^35^. Especially butyric acid has been shown to induce severe cytotoxic effects, followed by propionic and valeric acid^37^. In fact, McKee et al. showed that the most virulent bacteria produced the highest concentrations of these cytotoxic metabolites out of all tested *P.* *gingivalis* strains^31^. Therefore, the increased production of volatile butyric acid in the presence of amoxicillin could be a counteraction by *P.* *gingivalis* to an environmental threat*.* One possible reason for the differences between other fatty acids and butyric acid is that *P.* *gingivalis* has several possible routes to produce butyric acid, while other fatty acids are mainly produced through acetyl coenzyme A (acetyl-CoA), as seen in Fig. 6. Shifts in glycolysis activity compared to amino acid utilization due to antibiotic stress could lead to differences in the VOC production. Fatty acids are common metabolites for many bacterial species, as well as, the human body, which makes them poor biomarkers individually. However, the combined changes in volatile fatty acid concentrations could be used for monitoring effects of antimicrobial treatment. Especially the relationship between butyric acid and the other fatty acids should be further investigated.

According to our results, the real-time production of volatile indole is higher, when amoxicillin is not present. However, when corrected with the cell density, the end-point concentration is higher in cultures treated with amoxicillin. These results support some of the earlier findings^27,28^. Indole is a product of tryptophan metabolism, as can be seen from Fig. 6. Indole production by various bacterial species has been studied extensively^28,41^. It has been connected, among other thing, to signaling, drug resistance and biofilm formation^41^. Han et al. showed that *E.* *coli* increases indole production in response to antibiotic treatment and that it improves cell survival^28^. Indole has also been shown to increase the drug resistance of bacteria that do not produce it by themselves^28,41^, and it has beneficial effects on human epithelial cells as well^28,41^. Consequently, indole seems to act as a way for cells to neutralize the effects of or to increase their resistance to a threat. Thus, volatile indole has potential as a marker for bacterial viability under antibiotic treatment. Ammonia, another product of tryptophan metabolism and other common metabolic routes, was also found to decrease slightly in the presence of amoxicillin. However, the PTR-MS signal for protonated ammonia is affected by contribution from the ion source, which distorts the recorded concentrations. For this reason, ammonia is not discussed further in this paper. Moreover, ammonia is a common constituent of human exhaled breath originating mainly from the urease activity of oral bacteria^42^, which makes ammonia an unideal bacterial biomarker.

We also found that the production of hydrogen sulfide by *P.* *gingivalis* is reduced by treatment with amoxicillin, which is evident both from the real-time measurements, and the cell density corrected end-point concentrations. The effect of amoxicillin on methanethiol production is less clear, with reduction evident only with large amoxicillin concentrations. However, according to the bacterial growth curves, the effect of 15 µg/mL of amoxicillin in our culture system inhibited growth only slightly, whereas 150 µg/mL decreased the growth by 70%. Consequently, the untreated cultures and the cultures treated with a smaller concentration of amoxicillin might have a similar capacity to produce certain compounds. Methanethiol production is most likely decreased by amoxicillin treatment, but not in the same capacity as hydrogen sulfide. *P.* *gingivalis* produces methanethiol enzymatically from l-methionine and hydrogen sulfide from l-cysteine^43^. In addition, l-cysteine can be turned into l-homocysteine and further to l-methionine, which produces methanethiol. Nakamura et al. showed that the methanethiol production by *P.* *gingivalis* is in fact not dependent on the presence of extracellular l-methionine, but production of hydrogen sulfide is dependent on l-cysteine^44^. Combining these findings to our results suggest that the decreased production of hydrogen sulfide in the presence of amoxicillin is most likely due to (1) the enzymatic activity shifting towards methanethiol production, or (2) increased utilization of extracellular l-cysteine leading to rapid depletion. The fact that methanethiol production in the presence of amoxicillin does not decrease in the same manner as hydrogen sulfide further corroborates this. Our results suggest that especially changes in the hydrogen sulfide production, or the ratio of hydrogen sulfide and methanethiol, are indicators for the viability of *P.* *gingivalis* in the presence of amoxicillin. Moreover, our results corroborate earlier findings of decreased hydrogen sulfide and methanethiol production in general, when bacteria are treated with antibiotics^21,25^.

The production of the discussed VOCs coincides with different bacterial growth phases, which is clear when combining the real-time production profiles of Figs. 2 and 3 and the corresponding growth curves (Supplementary Information Fig. S1). Indole and butyric acid are produced throughout the bacterial growth, while the other short- and medium-chain fatty acids are released in the later stationary and death phases, when the cells begin to lyse. Methanethiol and hydrogen sulfide are produced actively in the exponential phase, but their levels decrease in the stationary phase. In the latter part of the culturing, the effect of cell death and the resulting release of VOCs can be observed also for methanethiol and hydrogen sulfide. According to these results, different bacterial growth phase could be monitored via the VOCs produced by the bacteria. For example, the combined production of volatile indole, hydrogen sulfide, and methanethiol could be used as indicators of active growth, while most of the detected short- and medium-chain fatty acids are linked to cell death.

### Effects of limiting supplemental hemin

To our knowledge, gas-phase studies investigating the effects of iron source restriction in bacteria are scarce or non-existent. Consequently, in this section we compare findings from relevant liquid-phase studies to our results with *P.* *gingivalis*. It was found by McKee et al. that short- to medium-chain fatty acid, such as butyric and propionic acids, were formed by *P.* *gingivalis* in higher amounts under hemin excess^31^. Acetic acid was the only fatty acid reportedly produced in higher amounts when supplemental hemin was not available. Our results corroborate these findings, albeit measured in gas-phase. We also found valeric acid, caproic acid, caprylic acid and capric acid from the headspace in small amounts. They were increased in hemin-rich conditions. As mentioned earlier, the virulence of *P.* *gingivalis* has been connected to cytotoxic compounds, such as butyric acid^31,34–37^. It has also been shown that the most virulent effects are achieved in hemin-rich conditions^31^. Consequently, the lack of an external iron source seems to decrease the virulence of *P.* *gingivalis* significantly, among other things, because of the decreasing production of short- and medium-chain fatty acids. Our results show that the lack of an external iron source decreased the production of these virulence factors to the level of the negative control, except for acetic acid. The collapsed medium-chain fatty acid production of *P.* *gingivalis* in combination with increase in acetic acid is a promising marker for shifted bacterial metabolism under environmental stress.

McKee et al. found that *P.* *gingivalis* used extracellular tryptophan in hemin-limited conditions in lesser amounts (25% consumed) than in hemin-rich conditions (50% consumed)^31^, which suggests a decreased production of indole in hemin-limited conditions. Our results from the headspace measurements corroborates this. In fact, volatile indole is decreased to the level of the negative control in our measurements when supplemental hemin is not available. Absence of indole is a clear indicator of divergence in the metabolism of *P.* *gingivalis* cultures without supplemental hemin compared to hemin-rich conditions. Especially, because no major differences were found between the bacterial growth in these conditions. A possible reason for the downregulation of tryptophan utilization when supplemental hemin is not available is, of course, the lack of iron. Some reports suggest a link between tryptophan metabolism and iron both in bacteria and humans^45,46^, but there is no evidence of this for *P.* *gingivalis*. Proteomics is required to investigate this further. Interestingly, the effects of hemin and amoxicillin are opposite according to our results. *P.* *gingivalis* produces more indole in response to antibiotic stress, but the indole production collapses when supplemental hemin is not present. This further suggests that the tryptophan pathway could be severely disturbed when enough iron is not available, while other stressors have a different effect.

As mentioned, methanethiol and hydrogen sulfide are produced from the enzymatic break-down of methionine and cysteine, though with different efficiencies and different extracellular requirements of these amino acids. We found the maximum levels of volatile methanethiol and hydrogen sulfide to be relatively similar with and without supplemental hemin, while differences were observed at the end of the culturing. According to McKee et al. cysteine was used effectively by *P.* *gingivalis* in hemin-limited conditions (73% consumed) and in slightly lower amounts in hemin-rich conditions (55% consumed)^31^. Hydrogen sulfide production depends on the amount of extracellular cysteine, which means that its effective utilization in hemin-limited conditions leads to maximum concentrations of hydrogen sulfide much earlier than in hemin excess. This is supported by our findings, where hydrogen sulfide is produced effectively in the first 20 h of culturing, after which it drops, when cysteine is depleted. In hemin excess, hydrogen sulfide is produced consistently throughout the culturing. Similar trend is observed for methanethiol, although it is not reportedly dependent on the amount of extracellular methionine^44^. According to McKee et al., methionine was only utilized by *P.* *gingivalis* under hemin excess (27% consumed)^31^. Perhaps *P.* *gingivalis* in unable to utilize extracellular methionine, when supplemental hemin is not available, and therefore, intracellular methionine is depleted during the bacterial growth. This would lead to the rapid decline in methanethiol production seen in our results. In hemin-rich conditions extracellular methionine is utilized, which leads to stable production of methanethiol throughout the culturing. The differences between methanethiol and hydrogen sulfide production by *P.* *gingivalis* cultures with and without supplemental hemin are not as clear as with fatty acids and indole. Also, these sulfur compounds do not react to supplemental hemin availability as strongly as to antibiotic stress.

While the bacterial load can affect how much VOCs are produced, it is not the only factor. Metabolic changes due to bacterial stress response or the need to utilize internal iron reservoirs can also affect the amount and composition of the VOCs produced. In order to survive, *P.* *gingivalis* might utilize intracellular ferritin or synthesize the porphyrin ring from related protoporphyrins in when hemin is not available, but those processes most likely require more energy than the processes in hemin excess^30,47^. Resulting changes in the energy metabolism of *P.* *gingivalis* may lead to changes in the VOC production as well, while the overall growth of the bacteria is not severely affected. In this work, no significant growth-related effect was observed by limiting supplemental hemin in the first passage, however, clear changes in the VOC production were achieved, as seen in Figs. 4 and 5. According to these results, *P.* *gingivalis* accommodates the lack of supplemental hemin by shifting its metabolism, which can be observed in the VOCs produced. Consequently, the discussed VOCs are markers for the stress response of *P.* *gingivalis*, which has not been previously demonstrated in vitro.

As can be seen from the growth-curves (Supplementary Information Fig.S4), the exponential and stationary phases of the bacterial growth are reached slightly later in cultures without supplemental hemin available. This phenomenon has also been observed in earlier studies and results most likely from the need of the cells to use intracellular iron reservoirs for growth^48,49^. As mentioned, this is likely more energy-consuming than utilizing extracellular hemin, which slows the growth slightly. Again, the connection between different bacterial growth phases and the production of certain VOCs is clear from real-time production profiles in Figs. 4 and 5, and the corresponding growth curves (Supplementary Information Fig. S4). Much like discussed in the previous section, indole is produced throughout the culturing, while hydrogen sulfide and methanethiol are more clearly linked to the exponential phase. The production of short- and medium-chain fatty acids coincides with the stationary phase, while some contribution could also result from the cell lysis.

## Conclusions

To our knowledge, only a few intervention studies have been done with any bacterial species regarding volatile metabolites^20–26,50^. Furthermore, our study is the first to investigate the effects of antibiotic treatment and supplemental hemin limitation on the volatile compounds produced specifically by the pathogenic oral anaerobe *P.* *gingivalis*. Our research is also one of the first to demonstrate how bacterial growth and metabolism can be examined in real-time via changes in their volatile fingerprint.

Our results show that the treatment of *P.* *gingivalis* in vitro with amoxicillin changes the composition of its volatile metabolites. Especially acetic, propionic, and valeric acids are decreased with antibiotic treatment, as are hydrogen sulfide and methanethiol. In contrast, indole and butyric acid are increased with amoxicillin treatment. We also show that the availability of supplemental hemin effects the volatile metabolites of *P.* *gingivalis* significantly. Indole production is virtually negated when supplemental hemin is not available, as is the production of most short- and medium-chain fatty acids. However, acetic acid is clearly increased in these conditions. The changes in volatile metabolites are most likely linked to disturbances in the bacterial metabolism, induced by the stress response to environmental threats. Limiting supplemental hemin affects the production of volatiles by changing the metabolism of the bacteria, whereas antibiotic treatment affects the production of volatile metabolites also via growth restriction. Moreover, the effects of hemin availability on the VOCs released by *P.* *gingivalis* are clearly stronger compared to the treatment with amoxicillin. This finding supports the reports of potential in using iron-binding compounds, such as lactoferrin, in the treatment of periodontitis^51–54^.

Our results suggest that changes in the volatile fingerprint of *P.* *gingivalis*, and bacteria in general, could be used in the future to estimate the effectiveness of antimicrobial treatment or as markers for bacterial growth. Most significantly, because these metabolites are volatile, they could be measured from samples such as human exhaled breath or saliva headspace, without the need for traditional bacterial culturing. This in turn, could provide new possibilities in rapid pathogen testing and noninvasive diagnostics.

## Supplementary Information

Supplementary Information.

## Data Availability

All relevant data analyzed during this study is included in this published article and its Supplementary Information files. Raw measurement data generated during the current study is available from the corresponding author on reasonable request.
