# Supplementary material for: Antibiotic treatment and supplemental hemin availability affect the volatile organic compounds produced by P. gingivalis in vitro
Source: Sci Rep. 2022 Dec 29;12:22534. doi: 10.1038/s41598-022-26497-0 (PMC9800405; doi:10.1038/s41598-022-26497-0)
Supplement: Supplementary file 1 — Supplementary Information. [file 41598_2022_26497_MOESM1_ESM.pdf]

Supplementary Information for "Antibiotic treatment and supplemental hemin availability affect the volatile organic compounds produced by *P. gingivalis in vitro*" by Roslund, K., Uosukainen, M., Järvik, K., Hartonen, K., Lehto, M., Pussinen, P., Groop, P.-H. and Metsälä, M.

	Page
Growth curves in different amoxicillin concentrations	1
Effect of amoxicillin in different stages of bacterial growth	1
Production profiles of additional fatty acids	1
Compounds identified from the headspace of *P. gingivalis* ATCC 33277	2
Example of a compound only produced by Tryptic Soy Broth	3
Growth curves with and without supplemental hemin	3

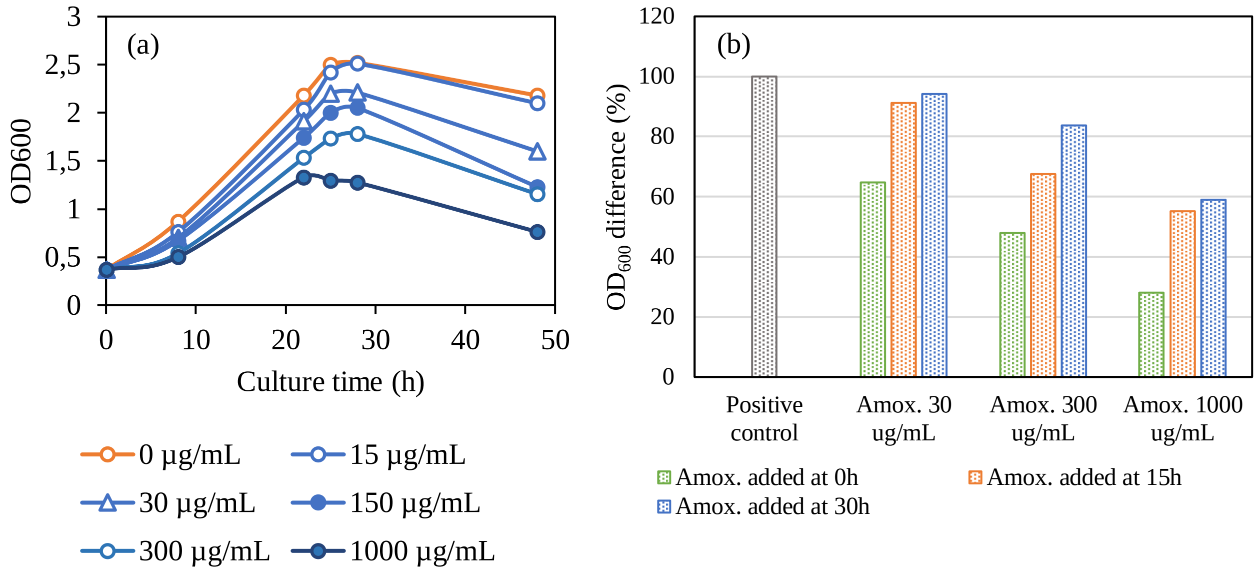

Fig.S1. (a) Growth curves of *Porphyromonas gingivalis* ATCC 33277 treated with different amounts of amoxicillin. Amoxicillin was added at the start of the culturing. **(b)** Effect of amoxicillin added in lag, exponential, and stationary phase (0, 15 and 30 h, respectively) on bacterial growth. The OD_{600} values were measured at 48 h.

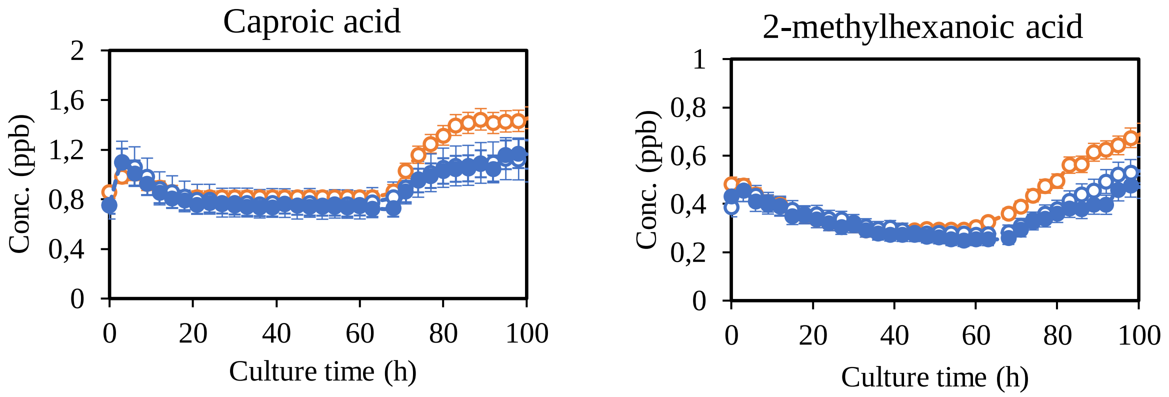

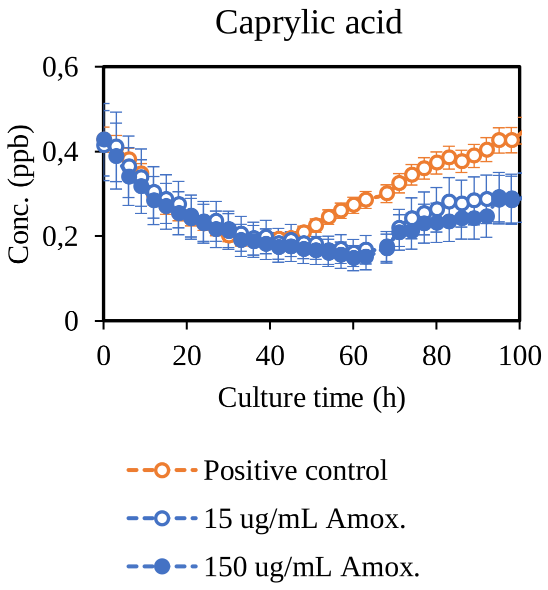

Fig.S2. Additional production profiles of volatile medium-chain fatty acids.

Table S1. Endpoint concentrations of additional fatty acids corrected with the corresponding OD600 value (mean ± SD, N=3).

	**Conc. (ppb)/OD_{600} at 90h**		
**Compound**	Positive control	15 µg/mL Amox.	150 µg/mL Amox.
Caproic acid	0.66±0.1	0.52±0.1	0.68±0.1
2-methylhexanoic acid	0.28±0.3	0.22±0.2	0.25±0.2
Caprylic acid	0.18±0.1	0.13±0.2	0.15±0.1

Table S2. Compounds identified from the headspace of *P. gingivalis* with GC-MS and PTR-ToF-MS. Hydrogen sulfide was only identified with PTR-ToF-MS. Protonated accurate and theoretical masses, as well as absolute mass differences are presented for all compounds without peak overlap with other signals. Absolute mass differences of more than 0.01 are marked in grey.

Mass (u)	Compound name	Molecular formula	Protonated Accurate Mass (u)	Protonated Exact Mass (u)	Absolute difference
34	*Hydrogen sulfide*	H_2S	*35.000*	*34.996*	*0.004*
48	Methanethiol	CH_4S	49.016	49.011	0.005
60	Acetic acid	$C_2H_4O_2$	61.034	61.029	0.005
74	Propanoic acid	$C_3H_6O_2$	75.050	75.045	0.005
88	Butyric acid*	$C_4H_8O_2$	89.065	89.060	0.005
88	2-Methylpropanoic acid (Isobutyric acid)	$C_4H_8O_2$	89.065	89.060	0.005
88	3-hydroxy-2-butanone (Acetoin)	$C_4H_8O_2$	89.065	89.060	0.005
94	Dimethylsulfone	$C_2H_6O_2S$	95.011	95.017	0.006
94	DMDS	$C_2H_6S_2$	94.9986	94.9989	0.0003
100	Hexanal	$C_6H_{12}O$	overlap	101.0966	
102	Pentanoic acid (Valeric acid) *	$C_5H_{10}O_2$	103.080	103.076	0.004
102	3-Methylbutanoic acid (Isovaleric acid)	$C_5H_{10}O_2$	103.080	103.076	0.004
108	Benzylalcohol	C_7H_8O	overlap	109.0653	
114	Heptanal	$C_7H_{14}O$	115.076	115.112	0.04
116	Hexanoic acid (Caproic acid)	$C_6H_{12}O_2$	117.093	117.092	0.001
117	Indole	C_8H_7N	118.071	118.066	0.005
126	DMTS	$C_2H_6S_3$	126.980	126.971	0.009
128	Octanal	$C_8H_{16}O$	overlap	129.1279	
130	2-methyl hexanoic acid	$C_7H_{14}O_2$	131.09	131.11	0.02
134	o-Cymene	$C_{10}H_{14}$	135.115	135.117	0.002
145	Octanoic acid (Caprylic acid)	$C_8H_{16}O_2$	145.124	145.123	0.001
147	6-metyyli-1,2,3,4-tetrahydroquinoline	$C_{10}H_{13}N$	overlap	148.1126	
156	Decanal	$C_{10}H_{20}O$	157.14	157.16	0.02
170	Undecanal	$C_{11}H_{22}O$	overlap	171.1749	
172	Decanoic acid (Capric acid)	$C_{10}H_{20}O_2$	173.152	173.154	0.003
184	Dodecanal	$C_{12}H_{24}O$	185.17	185.19	0.02

*The PTR-MS signals for butyric and valeric acid overlap with acetoin/isobutyric acid and isovaleric acid signals, respectively. However, in the GC-MS measurements the signal intensities for butyric and valeric acids are significantly larger, and therefore, they are suspected to be the main contributors to the PTR-MS signal as well.

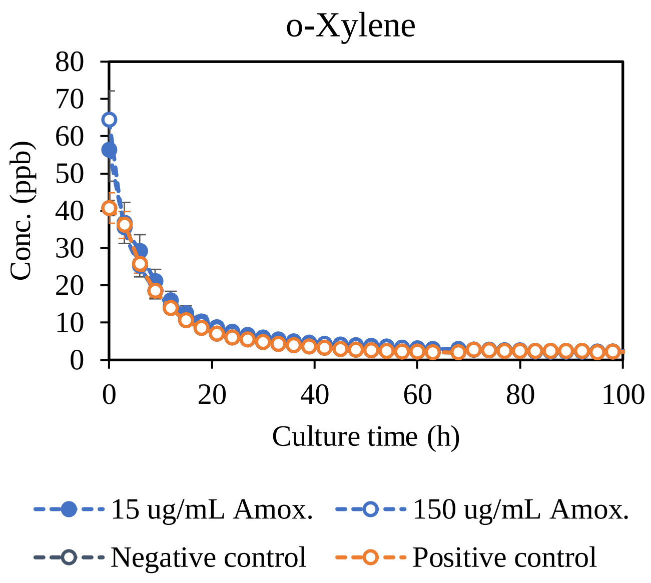

Fig. S3. Example of a production profile for a compound only produced by the nutrient broth, not by *P. gingivalis*.

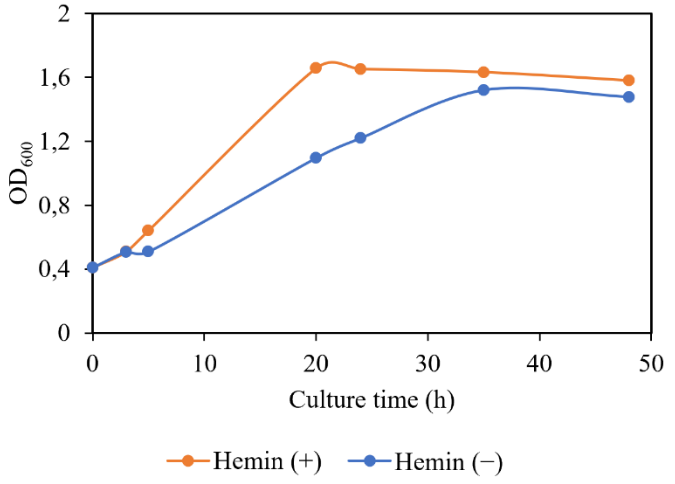

Fig. S4. Example of growth curves of *Porphyromonas gingivalis* ATCC 33277 in first passage, with and without hemin added to the growth medium (TSB).
